# Demographic Determinants and Geographical Variability of COVID-19 Vaccine Hesitancy in Underserved Communities: Cross-sectional Study

**DOI:** 10.2196/34163

**Published:** 2023-04-27

**Authors:** Jennifer L Matas, Latrice G Landry, LaTasha Lee, Shantoy Hansel, Makella S Coudray, Lina V Mata-McMurry, Nishanth Chalasani, Liou Xu, Taylor Stair, Christina Edwards, Gary Puckrein, William Meyer, Gary Wiltz, Marian Sampson, Paul Gregerson, Charles Barron, Jeffrey Marable, Olakunle Akinboboye, Dora Il'yasova

**Affiliations:** 1 National Minority Quality Forum Washington, DC United States; 2 University of Pennsylvania Philadelphia, PA United States; 3 Dana Farber Cancer Institute Boston, MA United States; 4 Harvard Medical School Boston, MA United States; 5 The George Washington University Washington, DC United States; 6 Morsani College of Medicine University of South Florida Tampa, FL United States; 7 Quest Diagnostics Secaucus, NJ United States; 8 Teche Action Clinic Franklin, LA United States; 9 Osceola Community Health Services Kissimmee, FL United States; 10 John Wesley Community Health Commerce, CA United States; 11 Aunt Martha’s Health and Wellness Olympia Fields, IL United States; 12 PrimaryOne Health Columbus, OH United States; 13 Queens Heart Institute Rosedale, NY United States; 14 Department of Family Medicine and Community Health School of Medicine Duke University Durham, NC United States

**Keywords:** COVID-19 vaccine, vaccine hesitancy, underrepresented in research, minority populations, federally qualified health center (FQHC), public health, COVID-19

## Abstract

**Background:**

COVID-19 hospitalizations and deaths disproportionately affect underserved and minority populations, emphasizing that vaccine hesitancy can be an especially important public health risk factor in these populations.

**Objective:**

This study aims to characterize COVID-19 vaccine hesitancy in underserved diverse populations.

**Methods:**

The Minority and Rural Coronavirus Insights Study (MRCIS) recruited a convenience sample of adults (age≥18, N=3735) from federally qualified health centers (FQHCs) in California, the Midwest (Illinois/Ohio), Florida, and Louisiana and collected baseline data in November 2020-April 2021. Vaccine hesitancy status was defined as a response of “no” or “undecided” to the question “Would you get a coronavirus vaccine if it was available?” (“yes” categorized as not hesitant). Cross-sectional descriptive analyses and logistic regression models examined vaccine hesitancy prevalence by age, gender, race/ethnicity, and geography. The expected vaccine hesitancy estimates for the general population were calculated for the study counties using published county-level data. Crude associations with demographic characteristics within each region were assessed using the chi-square test. The main effect model included age, gender, race/ethnicity, and geographical region to estimate adjusted odds ratios (ORs) and 95% CIs. Interactions between geography and each demographic characteristic were evaluated in separate models.

**Results:**

The strongest vaccine hesitancy variability was by geographic region: California, 27.8% (range 25.0%-30.6%); the Midwest, 31.4% (range 27.3%-35.4%); Louisiana, 59.1% (range 56.1%-62.1%); and Florida, 67.3% (range 64.3%-70.2%). The expected estimates for the general population were lower: 9.7% (California), 15.3% (Midwest), 18.2% (Florida), and 27.0% (Louisiana). The demographic patterns also varied by geography. An inverted U-shaped age pattern was found, with the highest prevalence among ages 25-34 years in Florida (n=88, 80.0%,) and Louisiana (n=54, 79.4%; *P*<.05). Females were more hesitant than males in the Midwest (n= 110, 36.4% vs n= 48, 23.5%), Florida (n=458, 71.6% vs n=195, 59.3%), and Louisiana (n= 425, 66.5% vs. n=172, 46.5%; *P*<.05). Racial/ethnic differences were found in California, with the highest prevalence among non-Hispanic Black participants (n=86, 45.5%), and in Florida, with the highest among Hispanic (n=567, 69.3%) participants (*P*<.05), but not in the Midwest and Louisiana. The main effect model confirmed the U-shaped association with age: strongest association with age 25-34 years (OR 2.29, 95% CI 1.74-3.01). Statistical interactions of gender and race/ethnicity with the region were significant, following the pattern found by the crude analysis. Compared to males in California, the associations with the female gender were strongest in Florida (OR=7.88, 95% CI 5.96-10.41) and Louisiana (OR=6.09, 95% CI 4.55-8.14). Compared to non-Hispanic White participants in California, the strongest associations were found with being Hispanic in Florida (OR=11.18, 95% CI 7.01-17.85) and Black in Louisiana (OR=8.94, 95% CI 5.53-14.47). However, the strongest race/ethnicity variability was observed within California and Florida: the ORs varied 4.6- and 2-fold between racial/ethnic groups in these regions, respectively.

**Conclusions:**

These findings highlight the role of local contextual factors in driving vaccine hesitancy and its demographic patterns.

## Introduction

Vaccine hesitancy has been defined as a “delay in acceptance or refusal of vaccination despite the availability of vaccination services” and is recognized among the 10 major public health threats by the World Health Organization (WHO) [1]. A recent review of the literature showed that vaccine hesitancy has become 1 of the key research topics [2]. The Centers for Disease Control and Prevention (CDC) published a map with the estimates of COVID-19 vaccine hesitancy, demonstrating its wide geographical variability [3]. Such variability emphasizes the importance of cultural, social, and economic contexts in driving COVID-19 vaccine hesitancy. It has been documented that COVID-19 vaccine hesitancy and vaccination rates have been low among racial/ethnic minority populations [4], even though these populations have been disproportionately affected by the pandemic, as shown by the rates of hospitalizations and deaths [5-8]. However, in contrast to the United States, a greater prevalence of COVID-19 vaccine hesitancy was not found among Black participants in the United Kingdom [9], emphasizing the importance of contextual cultural and socioeconomic factors.

The focus of our research is underserved communities in different geographical regions of the United States with vastly different racial/ethnic, cultural, and socioeconomic contexts. People in historically underserved communities have a higher proportion of racial/ethnic minorities, groups that are disproportionally affected by COVID-19 [5-8]. Moreover, people in underserved communities are at higher risk of more severe COVID-19 outcomes because of a greater prevalence of comorbidities associated with severe COVID-19 [10]. Understanding the variability in vaccination interest within and between minority and underserved communities is essential as effective vaccine administration policies and interventions require a nuanced understanding of the obstacles [11]. To date, few studies of COVID-19 vaccine hesitancy have reported estimates from underserved communities; this includes studies conducted in Delaware, North Carolina, and Monterey County, California [12-14]. Two studies were conducted in the year 2020, one study in North Carolina and one in California. The study in North Carolina classified overall 69% of respondents as vaccine hesitant, with the highest prevalence among White (74%), followed by Black (62.7%) and Hispanic (59.5%) participants. The study in Monterey County, California, included Hispanic farmworkers and reported 48.4% “not extremely likely to get vaccinated.” The study in Delaware was conducted in March-May 2021 and reported an overall 42% vaccine hesitancy prevalence, with 45% of Black participants expressing vaccine hesitancy. These previous studies also demonstrate that vaccine hesitancy is likely to be driven by contextual factors specific to geographic regions.

Here, we present the results from a large Minority and Rural Coronavirus Insights Study (MRCIS; N=3462) conducted in November 2020-March 2021 among underserved racially/ethnically diverse communities across 4 geographical US regions. The MRCIS is a multisite, community-based, epidemiologic investigation of the social and structural determinants of health, clinical, environmental, and genetic factors associated with the COVID-19 pandemic in minority and rural communities in the United States. Our objectives are to describe the demographic determinants of COVID-19 vaccine hesitancy within each geographical region and compare the estimates of vaccine hesitancy in these underserved communities with those expected for the general population in their respective local regions.

## Methods

### Study Design

This cross-sectional analysis of COVID-19 vaccine hesitancy prevalence was conducted using the baseline data collected by the MRCIS. A total of 3735 participants were enrolled, 155 (4.1%) participants were not qualified to participate (under the age of 18 years), and 29 (0.8%) participants were removed from the study due to missing informed consent, resulting in a study population of 3551 (95.1%) participants. The analytical sample included 3491 (98.3%) participants after the exclusion of 60 (1.7%) participants, who responded to the vaccine hesitancy question as “N/A” or left that question blank. As the last step of the analysis, we excluded 29 participants who were erroneously enrolled twice with a separate ID; this exclusion yielded a final analytical sample of 3462.

### Study Participants

In November 2020, the National Minority Quality Forum (NMQF) launched the MRCIS, a prospective longitudinal investigation of risk and socioeconomic factors associated with the disproportionate impact of COVID-19 on minority and rural communities. Federally qualified health centers (FQHCs), funded through the Health Resources & Services Administration (HRSA), were invited to partner with the MRCIS as community-based health care providers. The FQHCs were established to operate in communities that are underserved and therefore have been underrepresented in research. Research sites were selected based on the high proportion of deaths in minority populations that they serve compared to the proportion of minorities in the state population. In total, 5 community health centers in 4 geographically diverse HRSA regions (regions 6, 4, 5, and 9) were selected to participate in the MRCIS. In the Southeast region, participants were recruited from FQHCs located in HRSA regions 4 and 6. Participants in HRSA region 4 were recruited from Osceola Community Health Services (OCHS), located in Kissimmee, Florida, and participants in HRSA region 6 were recruited from the Teche Action Clinic (TAC), located in Franklin, Louisiana. In the Southwest region, HRSA region 9, participants were recruited from the John Wesley County Hospital (JWCH), an FQHC located in Los Angeles, California. In HRSA region 5, recruitment occurred at 2 FQHCs: Aunt Martha (AM) in Olympia Fields, Illinois, and Primary One (PO) in Columbus Ohio.

Volunteers were recruited from a convenience sample of adults (age≥18 years). Recruitment strategies varied by site and included 1 or more of the following methods: (1) in-person recruitment at the site; (2) in-person recruitment at study centers set up in the communities, including the recreational center, a low-income housing complex, the fire department, and homeless shelters; and (3) advertising through flyers distributed to the community. Participants did not receive any compensation for participation in this study.

### Ethical Considerations

At enrollment, participants completed an informed consent form and a baseline survey. The protocol was reviewed and approved by the WIRB-Copernicus Group Institutional Review Board (WCG IRB; #1292174). Each participant received a unique study ID, and the identifying information was removed from the data set for analysis to maintain patient privacy and confidentiality.

### Data Collection and Outcome Definition

The data used in this analysis were collected in the MRCIS using a baseline survey, including self-reported age, gender, race, and ethnicity. The question for reporting race was the following: “Select all that apply for race: American Indian/Alaskan Native, Asian, Black/African American/Native Hawaiian/Other Pacific Islander, White, Other, and Prefer Not to Answer.” The options for the ethnicity question were the following: “Hispanic or Latino,” “Not Hispanic or Latino,” and “Prefer not to answer.” We used the race and ethnicity questions to develop combined race/ethnicity categories. Although participants were allowed to select more than 1 category for the race, no one in our sample reported multiple races. All individuals who self-reported Hispanic/Latino as their ethnicity were characterized as Hispanic/Latino regardless of what they indicated as their race. We developed 4 mutually exclusive racial/ethnic categories as follows: Hispanic/Latino, non-Hispanic Black, non-Hispanic White, and non-Hispanic other.

Participants were asked, “Would you get a coronavirus vaccine if it was available,” with the option to answer “yes,” “no,” or “undecided.” According to the WHO definition [1], vaccine hesitancy was defined as a response of “no” or “undecided.” The responses were recorded from November 2020 to March 2021.

### Statistical Analysis

#### Vaccine Hesitancy Prevalence by Demographic Characteristics and Geographical Region

Self-reported age was categorized into the following age groups: 18-24, 25-34, 35-44, 45-54, 55-64, and ≥65 years. Race and ethnic information were categorized as noted before (Hispanic/Latino, non-Hispanic Black/African American, non-Hispanic White, and non-Hispanic other). Gender was characterized as female, male, and other (n=3). Due to a small sample size, the “Other” gender category was excluded from the regression analysis that adjusted for gender as a confounder. Descriptive statistics, including counts and proportions comparing individual-level factors in the overall study population and by vaccine hesitancy (not hesitant vs hesitant), were calculated. The strength of the adjusted associations of vaccine hesitancy prevalence with the demographic characteristics and geographical regions was assessed in logistic regression models by calculating odds ratios (ORs) and their 95% CIs. The main effect model included age (6 categories), gender (females vs males), race/ethnicity (4 categories), and HRSA site (4 sites). The reference groups for age, gender, race/ethnicity, and HRSA site were individuals aged 65 years and above, males, non-Hispanic White, and California, respectively.

#### Observed and Expected Vaccine Hesitancy Prevalence

The observed vaccine hesitancy prevalence and corresponding 95% CIs were calculated for each of the study sites. The expected vaccine hesitancy was calculated based on the county-level estimates reported by the CDC [3].

Each MRCIS participant was assigned to a residential county based on the reported residential zip code and the US Department of Housing and Urban Development (HUD) crosswalk files [15]. These crosswalk files are derived directly from the United States Postal Service (USPS) and are updated quarterly reflecting changes in zip code configurations. Converting the MRCIS participant zip code to residential counties enabled us to compare the MRCIS prevalence of vaccine hesitancy to the national estimates reported by the CDC [3]. National COVID-19 vaccine hesitancy data were downloaded directly from the CDC website. The CDC’s outcome definition for vaccine hesitancy was derived from the US Census Bureau’s Household Pulse Survey (HPS), which asked the following survey question: “Once a vaccine to prevent COVID-19 is available to you, would you…get a vaccine?” Participants were given the following response options: (1) “definitely get a vaccine,” (2) “probably get a vaccine,” (3) “unsure,” (4) “probably not get a vaccine,” and (5) “definitely not get a vaccine.” The estimate of vaccine hesitancy we used was “hesitant or unsure,” which directly compares with our definition of vaccine hesitancy [3].

For each region, the expected number of hesitant subjects was calculated by multiplying the number of MRCIS participants per county by the CDC’s reported vaccine hesitancy prevalence. For example, 930 participants resided in Los Angeles County, California, where the vaccine hesitancy was estimated by the CDC as 9.6%, so the expected number of vaccine-hesitant participants was 89.2. With 27.1% vaccine hesitancy estimates for St Mary Parish, Louisiana, among 882 participants, 239 were expected to be hesitant. The expected number of participants was summed across each study site, and their percentage (ie, percentage of the participants at the site) was calculated. In addition to the expected vaccine hesitancy prevalence, we provided a county-based range for estimated vaccine hesitancy in each MRCIS site.

The assessment of COVID-19 vaccine hesitancy was conducted by the CDC and our study in different periods (mid-2021 vs November 2020-March 2021). Based on the previously published time trends of COVID-19 vaccine hesitancy, it is likely that our expected estimates are approximately 1/3 greater compared to the CDC’s reported ones [16]. Therefore, we presented time-adjusted expected prevalence in the general population in the counties of our participants' residences.

#### Demographic Patterns of Vaccine Hesitancy Prevalence by Geographical Region

**Figure 1:**
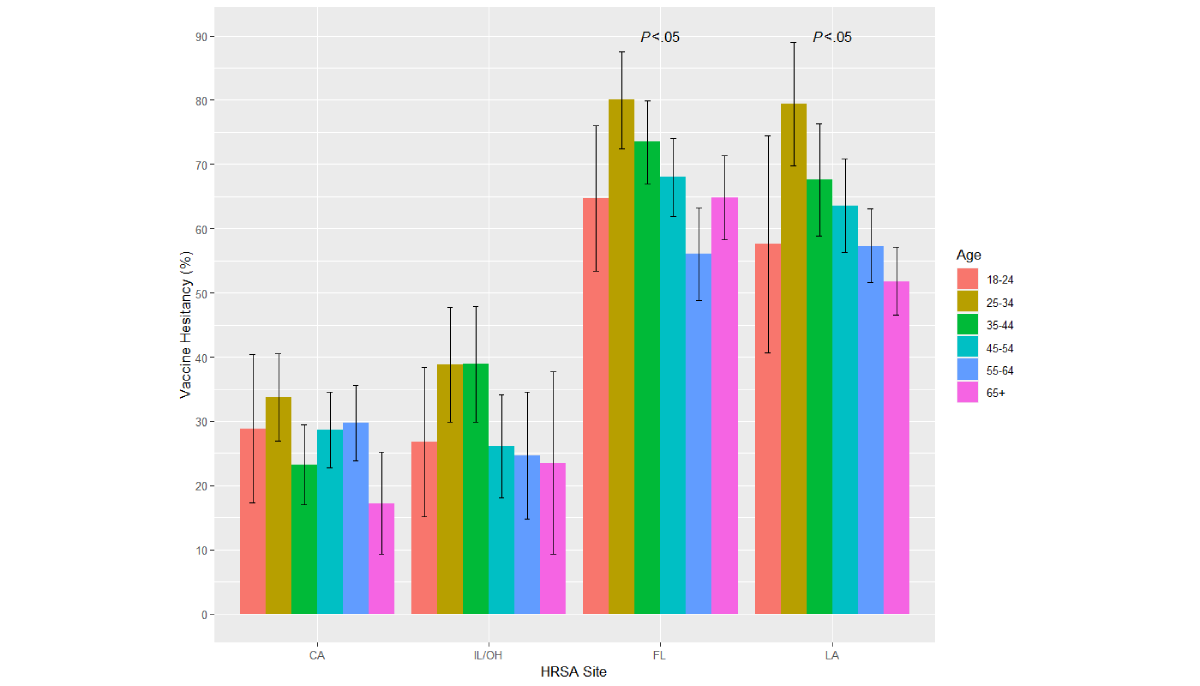
Vaccine hesitancy by age and geographical location, the MRCIS (2020-2021). CA: California; FL: Florida; HRSA: Health Resources & Services Administration; IL: Illinois; LA: Louisiana; MRCIS: Minority and Rural Coronavirus Insights Study; OH: Ohio.

**Figure 2:**
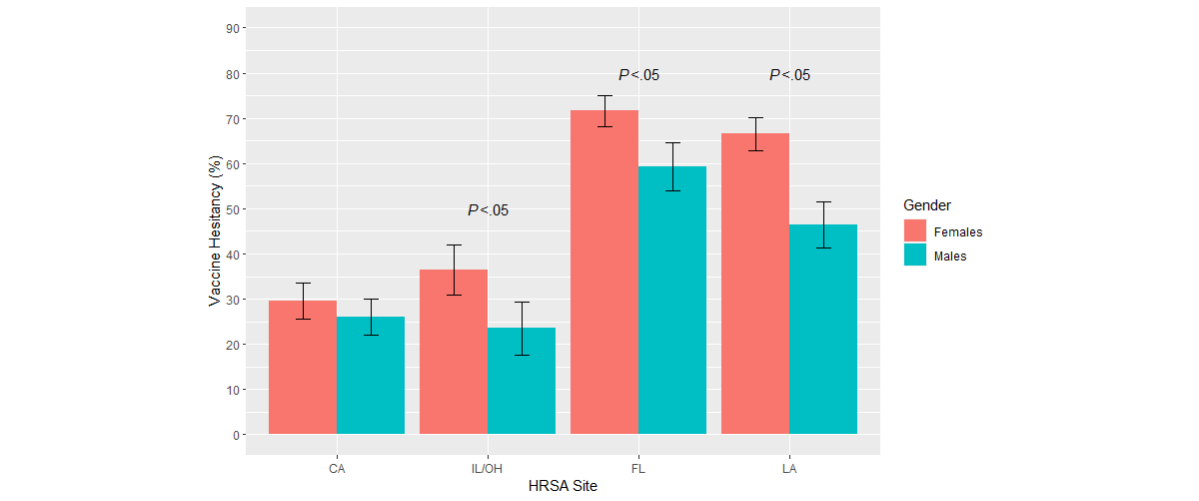
Vaccine hesitancy by gender and geographical location, the MRCIS (2020-2021). CA: California; FL: Florida; HRSA: Health Resources & Services Administration; IL: Illinois; LA: Louisiana; MRCIS: Minority and Rural Coronavirus Insights Study; OH: Ohio.

**Figure 3:**
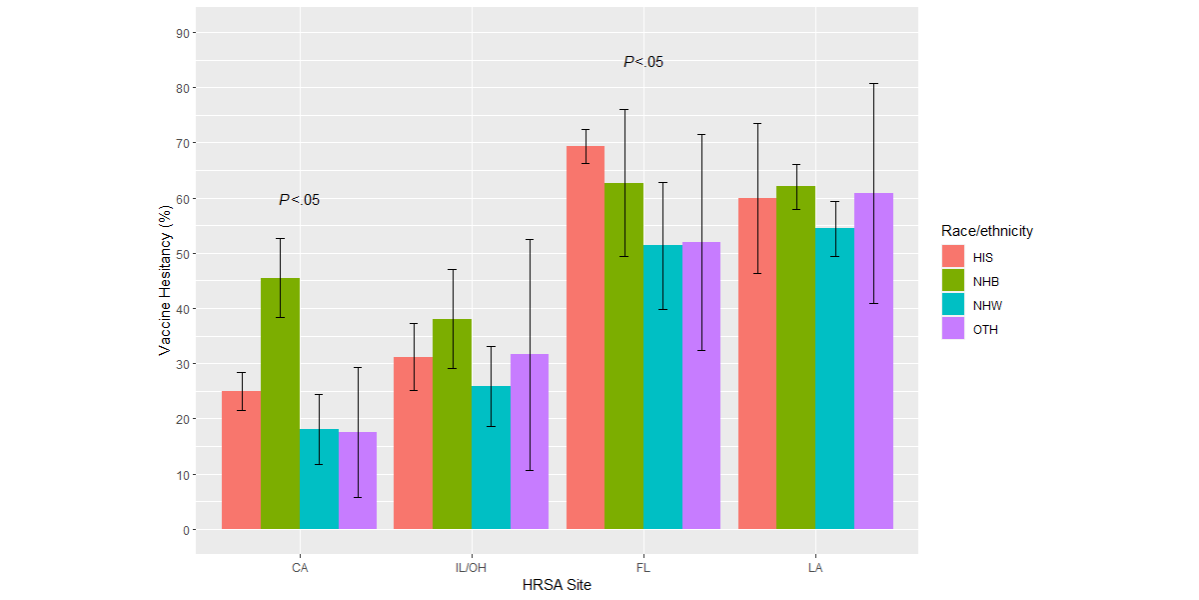
Vaccine hesitancy by race ethnicity and geographical location, the MRCIS (2020-2021). CA: California; FL: Florida; HIS: Hispanic/Latino; HRSA: Health Resources & Services Administration; IL: Illinois; LA: Louisiana; MRCIS: Minority and Rural Coronavirus Insights Study; NHB: non-Hispanic Black/African American; NHW: non-Hispanic White; OH: Ohio; OTH: non-Hispanic other.

Age, gender, and the race/ethnicity patterns of vaccine hesitancy were explored in descriptive analyses (Figures 1-3). The Pearson chi-square test was performed to determine the statistical significance of each demographic pattern within each study site. As this crude analysis strongly suggested the modification of the demographic patterns by geographical region, multivariable logistic regression models were performed to evaluate the interactions between each of the demographic covariates and the study site in separate logistic regression models. In the model exploring the interaction between age and geographical region, individuals in California aged 55 years and above served as the common reference group. In this model, we collapsed the 2 oldest categories (55-64 and ≥65 years) because the main effects model detected similar adjusted associations of these age categories with vaccine hesitancy prevalence. In the model exploring the interaction between gender and geographical region, males in California served as the common reference group. Lastly, non-Hispanic White participants in California served as the common reference group in the model exploring the interaction between race/ethnicity and geographical region. A type 1 error rate of 5% was used in the entire analysis.

Akaike information criterion (AIC) statistics were used to assess model fitness, in which lower AIC estimates indicated improved model fitness. We reported the following AIC statistics:

- Main effects multivariable logistic regression model AIC=4218.985
- Multivariable logistic regression model with the interaction between age and region AIC=4227.904
- Multivariable logistic regression with the interaction of gender and region AIC=4213.966
- Multivariable logistic regression with the interaction of race/ethnicity and region AIC=4200.983

As demonstrated by the AIC, the interaction terms between age and region did not improve the model, with the interaction term *P* value of .32.

All analyses were conducted in SAS version 9.4 (SAS Institute) and R version 4.2.1 (Comprehensive R Archive Network).

## Results

### Participant Details

Our study population was diverse, including females (n=2076, 60.0%) and males (n=1379, 39.8%) aged 18-94 years. The 3 major racial/ethnic groups were well represented, with the largest group presented by Hispanic participants (n=1684, 48.6%), followed by non-Hispanic Black/African American (n=896, 25.9%) and non-Hispanic White (n=740, 21.4%) participants. Other non-Hispanic racial/ethnic groups were presented by 107 (3.1%) participants; due to the small sample size, this demographic group was not further stratified (Table 1). Among females, 781/2076 (37.6%) were considered as being of reproductive age (18-44 years).

In this study population, vaccine hesitancy exceeded 50% among the following demographic groups: females, participants 25-44 years of age, and non-Hispanic Black/African American participants (Table 1). Overall, no linear trend was apparent in vaccine hesitancy with the increase in age. In relation to geography, a striking variability of hesitancy prevalence was observed: the highest prevalence was found in Florida (653/1680, 38.9%) and Louisiana (597/1680, 35.5%); see Table 1. The differences between the highest (Florida) and the lowest (California) hesitancy prevalence were greater than 2-fold (Table 1). Because of this strong geographic variability, we explored whether the observed geographic differences were similar to what would be expected in the general population within the same geographic areas.

The range of vaccine hesitancy in the general population is presented for the counties where our participants resided (Table 2). The expected vaccine hesitancy in the general population overall resembled the geographical pattern that we observed in the underserved communities, that is, lower in California and the Midwest (Illinois/Ohio) compared to Florida and Louisiana. Considering the expected time trend, that is, approximately 1/3-fold reduction in hesitancy by mid-2021 (period of estimates from the CDC) as opposed to 2020-early 2021, when our study was conducted, we adjusted the expected vaccine hesitancy prevalence as presented in Table 2. Even after such an adjustment, the observed vaccine hesitancy prevalence in the underserved population tended to be greater than expected in the general population (Table 2). These results further emphasized the importance of assessing vaccine hesitancy in the underserved population.

Examination of the crude (unadjusted) prevalence estimates for vaccine hesitancy showed that their demographic patterns vary between geographical regions. There was no clear age pattern among the California and Midwest participants (Figure 1). In other regions, a trend for greater hesitancy among participants aged 25-44 years was evident (*P*<.05); see Figure 1. Greater vaccine hesitancy among females was noticeable in all regions but was more pronounced (*P*<.05) in Florida, Louisiana, and the Midwest (Figure 2). Racial/ethnic differences were detected in California and Florida (*P*<.05) but not in Louisiana and the Midwest (Figure 3).

The adjusted estimates of the associations between vaccine hesitancy prevalence and both demographic and geographical characteristics (main effect model, Table 3) followed a pattern similar to the observations presented in Table 1. An inverted U-shaped correlation was found between vaccine hesitancy and age. Specifically, vaccine hesitancy was less associated with the younger and older age groups, whereas the association with age was 1.4-2.3-fold greater among adults aged 25-54 years compared to older adults aged ≥65 years (Table 3). Within the 25-54-year age group, there was a clear trend of the inverse association with age, with vaccine hesitancy declining among participants older than 25-34 years (Table 3). Females were approximately 1.7-fold more likely to express vaccine hesitancy compared to males. We hypothesized that vaccine hesitancy would be even more strongly associated with the female gender among females of reproductive age and tested the interaction between gender and age categorized as reproductive (18-44 years) and older (≥45 years); this model with age-gender interaction did not reveal differences in the association with gender by age. Non-Hispanic Black/African American and Hispanic participants had 1.8- and 1.2-fold greater odds of vaccine hesitancy compared to non-Hispanic White participants, respectively (Table 3). Overall, the magnitude of the strongest associations between demographic characteristics and vaccine hesitancy clustered around ORs of 2. Much stronger associations were found with geographical regions. Compared to California, even after adjustment for age, gender, and race/ethnicity, the odds of vaccine hesitancy were almost 6-fold greater in Florida and 4-fold greater in Louisiana (Table 3). Thus, the main effect analysis demonstrated that the geographical region has the strongest association with vaccine hesitancy.

**Table 1:** Population characteristics and vaccine hesitancy status in the MRCIS^a^ (2020-2021).

Characteristics			Participants (N=3462), n (%)		Vaccine hesitancy	
					Not hesitant (n=1782, 51.5%), n (%)	Hesitant (n=1680, 48.5%), n (%)
**Age (years)**						
	<25	216 (6.2)		121 (6.8)		95 (5.6)
	25-34	478 (13.8)		229 (12.9)		249 (14.8)
	35-44	586 (16.9)		292 (16.4)		294 (17.5)
	45-54	737 (21.3)		381 (21.4)		356 (21.2)
	55-64	775 (22.4)		421 (23.6)		354 (21.1)
	≥65	665 (19.2)		334 (18.7)		331 (19.7)
	Missing	5 (0.1)		4 (0.2)		1 (0.1)
**Gender**						
	Female	2076 (60.0)		937 (52.6)		1139 (67.8)
	Male	1379 (39.8)		840 (47.1)		539 (32.1)
	Other	3 (0.1)		3 (0.2)		0
	Missing	4 (0.1)		2 (0.1)		2 (0.1)
**Race/ethnicity**						
	Hispanic/Latino	1684 (48.6)		869 (48.8)		815 (48.5)
	Non-Hispanic Black/African American	896 (25.9)		398 (22.3)		498 (29.6)
	Non-Hispanic White	740 (21.4)		429 (24.1)		311 (18.5)
	Non-Hispanic other	107 (3.1)		67 (3.8)		40 (2.4)
	Missing	35 (1.0)		19 (1.1)		16 (1.0)
**HRSA^b^ site**						
	California	971 (100.0)		701 (39.3)		270 (16.1)
	Florida	971 (100.0)		318 (17.8)		653 (38.9)
	Midwest (Illinois/Ohio)	510 (100.0)		350 (19.6)		160 (9.5)
	Louisiana	1010 (100.0)		413 (23.2)		597 (35.5)

^a^MRCIS: Minority and Rural Coronavirus Insights Study.

^b^HRSA: Health Resources & Services Administration.

**Table 2:** Vaccine hesitancy estimates in the general population, as reported by the CDC^a^ (2021), and in the underserved population, as observed by the MRCIS^b^ (2020-2021).

Sites of data collection	Number of counties in each HRSA^c^ site	CDC vaccine hesitance prevalence estimates in HRSA site counties	Expected vaccine hesitancy prevalence^d^ (adjustment for time trend)^e^	MRCIS observed prevalence of vaccine hesitancy (95% CI)
California	6	8.2%-13.3%	9.7% (12.9%)	27.8% (25%-30.6%)
Midwest (Illinois/Ohio)	23	10.4%-23.7%	15.3% (20.3%)	31.8% (27.7%-35.8%)
Florida	8	16.0%-20.4%	18.2% (24.2%)	67.3% (64.3%-70.2%)
Louisiana	11	21.7%-27.9%	27.0% (36%)	59.1% (56.1%-62.1%)

^a^CDC: Centers for Disease Control and Prevention.

^b^MRCIS: Minority and Rural Coronavirus Insights Study.

^c^HRSA: Health Resources & Services Administration.

^d^Based on the CDC estimates for the counties of the participant’s residence (2021).

^e^Based on the previously published time trend estimates, the expected prevalence of vaccine hesitancy decreased by 1/3 between 2020 and 2021; therefore, we increase the estimate for the expected vaccine hesitancy to reflect what would be expected at the time of our survey.

**Table 3:** Association of vaccine hesitancy with demographic characteristics and geographical location, as observed by the MRCIS^a^ (2020-2021).

Main Effect Model		Adjusted OR^b^ (95% CI)
**Age (years)**		
	18-24	1.33 (0.94-1.87)
	25-34	2.29 (1.74-3.01)
	35-44	1.59 (1.24-2.04)
	45-54	1.38 (1.10-1.75)
	55-64	1.12 (0.89-1.40)
	≥65	Reference
**Gender**		
	Female	1.67 (1.44-1.94)
	Male	Reference
**Race/ethnicity**		
	Hispanic/Latino	1.24 (0.99-1.55)
	Non-Hispanic Black	1.79 (1.45-2.21)
	Non-Hispanic White	Reference
	Non-Hispanic other	0.93 (0.59-1.46)
**HRSA^c^ site**		
	California	Reference
	Midwest (Illinois/Ohio)	1.11 (0.87-1.42)
	Florida	5.81 (4.72-7.16)
	Louisiana	4.03 (3.20-5.08)

^a^MRCIS: Minority and Rural Coronavirus Insights Study.

^b^OR: odds ratio.

^c^HRSA: Health Resources & Services Administration.

We further explored the adjusted associations between each demographic characteristic and vaccine hesitancy, considering the geographical differences (models with interaction terms, Tables 4 and 5). These models showed significant interactions (*P*<.05 for the interaction term) between the geographical region and 2 demographic characteristics, namely gender and race/ethnicity, not with age. The adjusted association with gender followed the same tendencies revealed by the crude analysis (Figure 2 and Table 4). Within each gender group, the association between the female gender and vaccine hesitancy was strongest in Florida, followed by Louisiana and the Midwest (Table 4). Within each region, the tendency of a stronger association with the female gender was obvious; however, only in Louisiana, the 95% CIs for the gender-specific estimates did not overlap, indicating sufficient precision to detect gender differences in this region.

The racial/ethnic differences in the adjusted estimates of vaccine hesitancy also followed the pattern found in the crude analysis (Figure 3). As compared to non-Hispanic White participants in California, all racial/ethnic groups in Florida and Louisiana had a greater association with vaccine hesitancy (Table 5). In California, non-Hispanic Black/African American participants clearly had greater odds of vaccine hesitancy compared to non-Hispanic White participants. However, in other regions, the likelihood that non-Hispanic Black participants would express vaccine hesitancy was similar to other racial/ethnic groups. The strongest association of vaccine hesitancy with being non-Hispanic White was observed in Louisiana, whereas the strongest association with being Hispanic was observed in Florida. Thus, in this study, the demographic patterns of vaccine hesitancy, especially racial/ethnic differences, strongly depended on local contextual factors defined by geographical region.

**Table 4:** Association between vaccine hesitancy and gender modified by geographical location, as observed by the MRCIS^a^ (2020-2021).

Gender	California, OR^b^ (95% CI)^c^	Midwest (Illinois/Ohio), OR (95% CI)^c^	Florida, OR (95% CI)^c^	Louisiana, OR (95% CI)^c^
Female	1.14 (0.86-1.52)	1.58 (1.14-2.17)	7.88 (5.96-10.41)	6.09 (4.55-8.14)
Male	Reference^d^	0.83 (0.56-1.23)	4.66 (3.41-6.35)	2.80 (2.03-3.85)

^a^MRCIS: Minority and Rural Coronavirus Insights Study.

^b^OR: odds ratio.

^c^Multivariable logistic regression models were adjusted for age and race/ethnicity.

^d^Males in California served as the common reference group.

**Table 5:** Association between vaccine hesitancy and race/ethnicity modified by geographical location, as observed by the MRCIS^a^ (2020-2021).

Race/ethnicity	California, OR^b^ (95% CI)^c^	Midwest (Illinois/Ohio), OR (95% CI)^c^	Florida, OR (95% CI)^c^	Louisiana, OR (95% CI)^c^
Hispanic	1.49 (0.92-2.41)	1.95 (1.15-3.30)	11.07 (6.93-17.70)	7.25 (3.50-15.02)
Non-Hispanic Black/African American	4.61 (2.71-7.84)	2.60 (1.44-4.69)	8.07 (3.90-16.67)	8.88 (5.48-14.37)
Non-Hispanic Whites	Reference^d^	1.53 (0.85-2.74)	5.38 (2.81-10.29)	6.68 (4.09-10.92)
Non-Hispanic other	0.99 (0.39-2.52)	1.84 (0.62-5.41)	5.63 (2.26-14.02)	8.32 (3.19-21.68)

^a^MRCIS: Minority and Rural Coronavirus Insights Study.

^b^OR: odds ratio.

^c^Multivariable logistic regression models were adjusted for age and gender.

^d^Non-Hispanic White participants in California served as the common reference group.

## Discussion

### Principal Findings

Our study of COVID-19 vaccine hesitancy in underserved populations revealed several important findings. We found profound differences in vaccine hesitancy between geographical regions (Table 1). These observed differences might be expected based on the estimates published by the CDC (Table 2). However, our estimates for the MRCIS population showed a clear tendency of greater vaccine hesitancy than expected in the general population. This finding is important because the underserved population is at a greater risk of worse outcomes of this infection [5-8]. Thus, our study highlights the need to clearly communicate the benefits and risks of the vaccine to this high-risk population.

Another important finding is the difference in demographic patterns of vaccine hesitancy by geographical region. Although in California, the greatest vaccine hesitancy was among non-Hispanic Black/African American participants, this finding cannot be extrapolated to Louisiana and Florida. Similarly, the highest hesitancy prevalence among Hispanic participants in Florida cannot be extrapolated to other regions. These findings suggest that local contextual factors are major contributors to vaccine hesitancy in contrast to demographic characteristics. Future studies of vaccine hesitancy and other health behaviors should consider the local specifics. Additionally, these findings could prove important to developing effective local public health initiatives as streamlined, catch-all approaches may lack the nuance and specificity needed to impact vulnerable communities.

Results of prior studies support our findings, emphasizing the importance of local contextual factors in driving racial/ethnic patterns of vaccine hesitancy. A prior study conducted in the underserved population of North Carolina found the highest prevalence among White (74%), followed by Black (62.7%) and Hispanic (59.5%) participants, whereas a study in the Delaware underserved community found the greatest hesitancy among Black participants (45%). The online study conducted by the market research firm YouGov [17] found a similar prevalence of vaccine hesitancy among Black and White participants in 2020, once again confirming that racial/ethnic patterns strongly depend on the study population examined and cannot be extrapolated from one population to another.

We found that vaccine hesitancy tended to be lower among participants younger than 25 years and older than 55 years in the Midwest, Florida, and Louisiana (Figure 1 and Table 3). Evaluating the interaction between age and geographical region strongly suggests that in this study, the age patterns adjusted for gender and race/ethnicity did not differ by geography. The highest vaccine prevalence and the strongest association with age were found among ages 25-34 years. Similarly, among US Facebook respondents in January-May 2021, this age group had the highest vaccine hesitancy prevalence [18]. Another study conducted for the New York City Transport Workers Union found that adults aged 50 years and above were less likely to be COVID-19 vaccine hesitant than their younger counterparts [19]. These investigations examined study populations different from this report both socially and geographically. Thus, the age pattern with the high prevalence of vaccine hesitancy among ages 25-34 years appears to be consistent and less sensitive to the local contextual factors as opposed to racial/ethnic patterns.

The tendency of females to be more likely vaccine hesitant was apparent in all geographical regions but especially pronounced in Louisiana (Figure 2 and Table 4). Similar results were found by the US Facebook study, with females being more likely hesitant compared to men [18]. In addition, the analysis of a nationally representative sample from the 2021 Household Pulse Survey, administered by the US Census Bureau, showed greater vaccine hesitancy among US women [20]. Similar to the age patterns, gender differences in vaccine hesitancy may not be as sensitive to the local contextual factors and, therefore, are more generalizable to different population segments in the United States compared to racial/ethnic differences.

### Limitations

There are several limitations to our study. Because the participants were drawn from volunteers and represented a convenience sample, the results may be liable to self-selection bias. To discuss whether such a bias potentially increased or decreased participation among the vaccine-hesitant individuals, we considered the major incentive for participation (ie, the availability of COVID-19 testing). Such an incentive suggests that volunteers were concerned about their infection status. However, neither the COVID-19 incidence rate nor mortality correlated with vaccine hesitancy worldwide [21]. Thus, it is unclear whether a potential self-selection bias was likely to increase or decrease participation among the hesitant subgroup, given the interest in the COVID-19 test. Because similar geographical patterns were found by the CDC data (Table 2), likely, self-selection did not distort the observed differences by geographical region. Similar age and gender patterns found in our and other studies suggest that either self-selection bias may have influenced all these studies or a potential self-selection bias did not distort these associations. Finally, self-selection bias could influence racial/ethnic patterns. However, the direction of the bias should have been specific to contextual local factors governing the observed geographical differences. Overall, even considering the likelihood of self-selection bias, our study clearly demonstrates the importance of local contextual factors in driving COVID-19 vaccine hesitancy. Another limitation of our study is the lack of additional data to further investigate the reasons for the revealed differences in vaccine hesitancy. Which local contextual factors are important remains unknown. We will focus our future investigations on answering this question.

### Conclusion

In summary, our findings strongly suggest that local contextual factors drive the overall level of vaccine hesitancy in different regions, as demonstrated by the low prevalence in all demographic groups in California compared to Florida and Louisiana. Importantly, in all geographical regions, our estimates of vaccine hesitancy in the underserved population tended to be greater than what was expected in the general population in those same regions. Future investigations on this topic should include recording reasons for vaccine hesitancy to gain further insight into this subject. The same vaccine hesitancy factors will likely play a significant role in curbing future epidemics through vaccinations.

## Abbreviations

AIC: Akaike information criterion
CDC: Centers for Disease Control and Prevention
FQHC: federally qualified health centers
HRSA: Health Resources & Services Administration
MRCIS: Minority and Rural Coronavirus Insights Study
OR: odds ratio
WHO: World Health Organization
